# Risk Factors for Physical Function Impairments in Postintensive Care Syndrome: A Scoping Review

**DOI:** 10.3389/fped.2022.905167

**Published:** 2022-06-17

**Authors:** Min Ding, Chunfeng Yang, Yumei Li

**Affiliations:** Department of Pediatric Intensive Care Unit, The First Hospital of Jilin University, Changchun, China

**Keywords:** children, postintensive care syndrome, physical function impairments, pediatric intensive care unit, risk factors

## Abstract

**Objective:**

Survivors of critical illness may experience short- and long-term physical function impairments. This review aimed to identify the risk factors for physical function impairments from the current literature.

**Data Sources:**

A systematic search of the PubMed, Embase, Web of Science, and Cochrane Library databases following the Preferred Reporting Items for Systematic Reviews and Meta-analyses extension for Scoping Reviews guideline was performed.

**Study Selection:**

The risk factors reported in all human studies reporting physical function impairments in children admitted to the pediatric intensive care unit (PICU) were reviewed and categorized. Two investigators independently screened, evaluated, and selected studies for inclusion. Data from eligible studies were extracted by one investigator, and another investigator reviewed and verified the data. A systematic narrative approach was employed to review and summarize the data.

**Results:**

A total of 264 studies were found to be eligible, with 19 studies meeting the inclusion criteria. Children admitted to the PICU experienced physical function impairments during their stay, which can last for years. The studies varied primarily in the measurement timing and tools used. The most frequently reported risk factors for physical function impairments were age, race or ethnicity, a pre-admission chronic condition, sex, disease severity, duration or the presence of mechanical ventilation, and admission diagnosis.

**Conclusions:**

Physical function impairments may be persistent in PICU survivors. To prevent these impairments in critically ill patients, pediatricians should pay attention to modifiable risk factors, such as the duration of mechanical ventilation. Future studies need to promote a combination of standardized measures for the detection and prevention of physical function impairments.

## Introduction

A widespread range of temporary or long-term impairments can develop during and after stay in an intensive care unit (ICU) (1). Impairments refer to new or worsened physical function, mental health, or cognitive function, and they are known as postintensive care syndrome (PICS) (1, 2). PICS is a new term introduced in 2010, and there is still no clear operational definition, pathophysiology, or specific blood or radiologic tests to diagnose it (3, 4). Nevertheless, more physical and psychological problems following ICU discharge have been reported due to the increased survival rate.

Over the past decades, physical impairments seem to outlast neuropsychologic impairments, and they lead to limitations in activities of daily living, fatigue, weakness, pain, and delayed return to work (5–7). For instance, limitations in global muscle strength, called ICU-acquired weakness (ICUAW), have now been widely studied in adults, and it is likely a primary cause of significant financial, social, and medical burden to ICU survivors and their families (8–10). Although PICS has been well-conceptualized in adults, little is known about PICS in children. To systematically identify PICS in children, the PICS in pediatrics (PICS-p) framework was developed in 2018 (11). Similar to adults, children admitted to the pediatric intensive care unit (PICU) are at risk for long-term morbidities, including physical function impairments and ongoing morbidity following discharge even if the child had been previously healthy (12–14). A prior cohort study found that PICU survivors had a high physical impairment rate of 85.1% at discharge and a high rate of 55.7% at 6 months following hospital discharge (15). A poor level of physical function may have a substantial impact on both future health and developmental trajectories (11, 16, 17). It is important to identify the risk factors for physical function impairments and optimize preventative action to reduce their incidence and promote recovery.

To more efficiently manage physical function impairments in PICU survivors, a clearer understanding of the risk factors is needed. This review aimed to summarize the risk factors for physical function impairments during hospitalization or post-PICU/hospital discharge. In addition, it can provide a basis for the development of relevant interventions.

## Materials and Methods

### Study Design

This was a systematic review to identify the risk factors for physical function impairments. A comprehensive study protocol was recommended by the Preferred Reporting Items for Systematic Reviews and Meta-analyses extension for Scoping Reviews guidelines (18). This protocol is provided as Supplementary Material 1.

### Inclusion and Exclusion Criteria

#### Inclusion Criteria

-Patients: PICU survivors (<18 years old).
-Exposures: Risk factors for physical function impairments.
-Comparators: No physical function impairments risk factors.
-Outcomes: Any assessment method of physical function impairments.
-Study design: Cohort, case–control, and cross-sectional studies.
-Journal articles published in English from the earliest database records to October 2021.

#### Exclusion Criteria

-Studies conducted in neonatal ICUs.
-Reviews, qualitative research (e.g., letters to the editor, editorials, and study protocols), and studies that had not been peer-reviewed (e.g., published abstracts, conference proceedings, or dissertations).
-Full texts were retrieved, and articles not meeting this definition of physical function (e.g., endocrine and cardiac function).
-Studies that did not assess the risk factors for physical function impairments as an outcome variable.
-Multidimensional measures of “quality of life (QoL),” which cover a variety of physical and psychosocial outcomes.

### Search Strategy and Selection

The literature search and selection process was performed based on the Preferred Reporting Items for Systematic Reviews and Meta-analyses flow diagram. The following literature databases were searched: PubMed, Embase, Web of Science, and Cochrane Library. A combination of MeSH terms and keywords was used for the search, and the detailed search strategy is provided in Supplementary Material 2. Two of the authors (MD and CY) reviewed all the identified records and conducted the entire process independently. They discussed all disagreements throughout the process until a consensus was reached.

### Data Extraction and Analyses

Data were extracted using a structured form designed by the authors. The following characteristics were extracted and collected from the 19 included studies: author, publication year, country, study design, study subjects, sample size, selection criteria (length of ICU stay), sex and mean age of participants, measurement tools, timing and number of measurements, participants' diseases, and risk factors for physical function impairments. Data were independently extracted by two researchers (MD and CY) and entered into an Excel spreadsheet after a third researcher (YL) confirmed agreement. Due to the heterogeneity of the studies, no statistical synthesis was conducted, and a narrative approach was employed instead to interpret the literature.

## Results

### Study Selection

**Figure 1:**
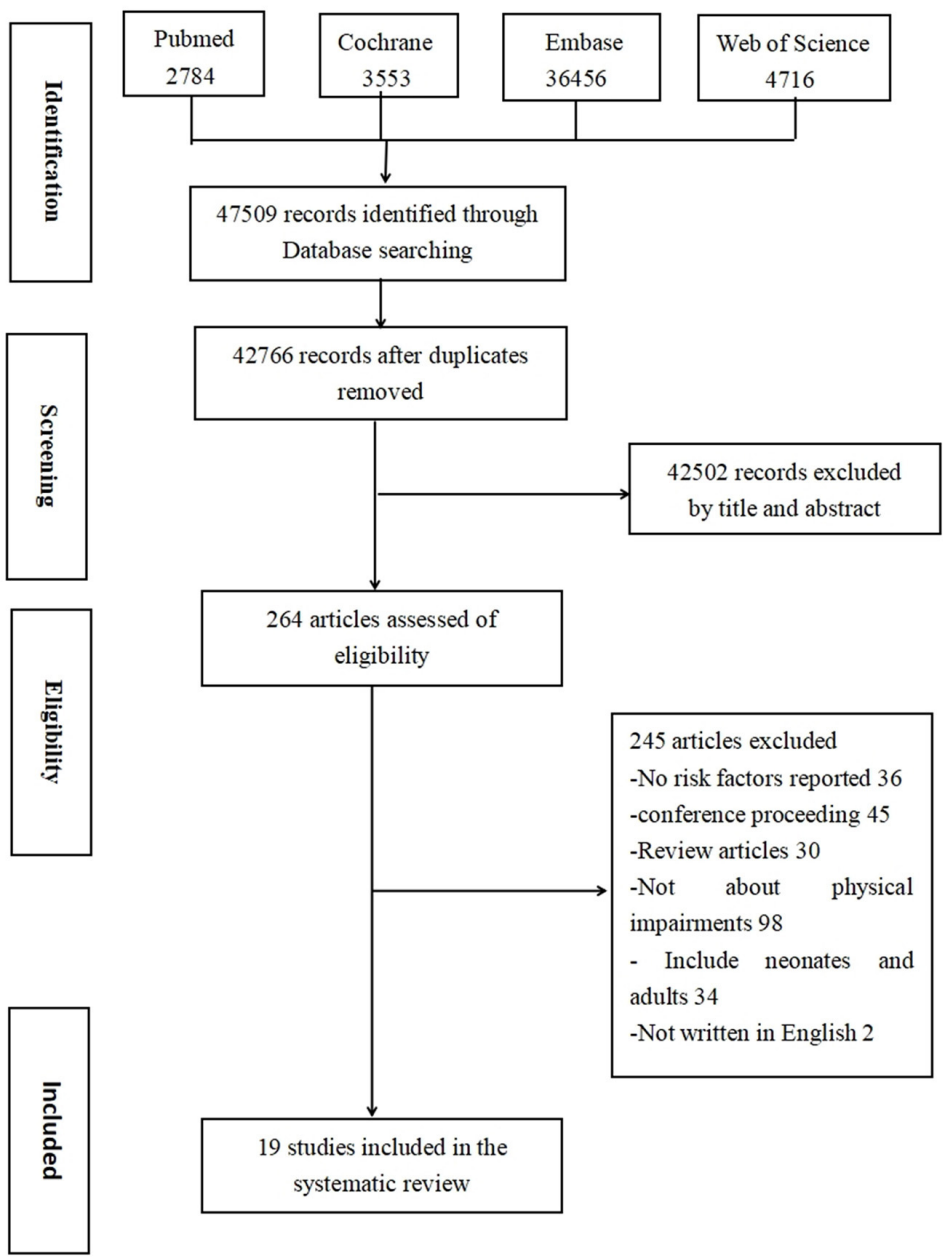
Flow diagram of study screening.

The screening and selection process is presented in Figure 1. A total of 47,509 articles were screened, and 42,766 articles were identified after eliminating duplicates. After researchers reviewed the title, abstract, and full text, 19 studies were selected for final inclusion.

### Study and Sample Characteristics

The characteristics of the 19 studies in the final analysis are presented in Table 1. The included studies were conducted in six different countries, and 13 studies (68.3%) were conducted in the United States. The publication years ranged from 1995 to 2021, and more than half of the studies (*n* = 14; 73.6%) were published between 2015 and 2021. Fourteen (73.6%) studies reported sex statistics and included more men than women. In these studies, the mean age of the participants was below 10 years old. Of the 19 included studies, 16 (84.2%) had unique samples involving 11,842 patients whose physical function impairments were reported. Of these, eight studies (50.0%) included heterogeneous samples and eight (50.0%) included homogeneous samples [acute respiratory failure (*n* = 1), sepsis (*n* = 3), hemophagocytic lymphohistiocytosis (*n* = 1), bronchiolitis (*n* = 1), congenital heart disease after open-heart surgery (*n* = 1), and bacterial meningitis (*n* = 1)]. A total of 8 (42.1%) studies included fewer than 100 patients. Multiple different instruments were used to report physical function impairments. A global physical function instrument, such as the Pediatric Overall Performance Category (POPC) (*n* = 5), was the most frequently used measurement instrument, followed by the Functional Status Scale (FSS) (*n* = 4). Other measurement instruments included biophysical instruments, such as X-ray (*n* = 1), ultrasound (*n* = 1), and physical function scales and scores (e.g., the Patient and Observer Scar Assessment Scale (POSAS) (*n* = 1), the Chalder Fatigue Scale (*n* = 1), the Sleep Disturbances Scale (*n* = 1), and the Medical Research Council score (*n* = 1)], and motor development instruments, such as the Denver Development Screening Test (*n* = 2), the Pediatric Evaluation of Disabilities Inventory Computer Adaptive Test (*n* = 1), and the Movement Assessment Battery for Children-II (MABC-II) (*n* = 1). Most studies found a morbidity rate of <60%.

**Table 1:** Study characteristics (*N* = 19).

**Characteristics**	**Categories**	***n* (%)**
Publication year	<2015	5 (26.4)
	2015–2018	7 (36.8)
	2019–2021	7 (36.8)
Publication country	USA	13 (68.3)
	Canada	2 (10.5)
	China	1 (5.3)
	Netherlands	1 (5.3)
	England	1 (5.3)
	Egypt	1 (5.3)
Study design	Cross-sectional studies	7 (36.8)
	Longitudinal studies	12 (63.2)
Sample size	<100	8 (42.1)
	100–200	6 (31.6)
	300–500	1 (5.3)
	≥500	4 (21.0)
Mean age of participants (years)	≤ 5	10 (52.6)
	5–10	6 (31.6)
	≥10	3 (15.8)
Male of participants	≤ 50%	5 (26.4)
	>50%	14 (73.6)
Inclusion criteria for ICU stay (hours)	≥24	4 (21.0)
	≥48	3 (15.8)
	Undecribed	12 (63.2)
Assessment tools (*n* = 20)	POPC	5 (25.0)
	FSS	4 (20.0)
	DDST	2 (10.0)
	Others^a^	9 (45.0)
Months from discharge to assessment (*n* = 25)	<1^b^	11 (44.0)
	1–3	2 (8.0)
	4–6	4 (16.0)
	7–12	2 (8.0)
	≥13	6 (24.0)
Diseases of samples	ABI^c^	5 (26.4)
	Sepsis	3 (15.8)
	ARF	3 (15.8)
	General PICU patients	4 (21.0)
	Others^d^	4 (21.0)
Number of PICUs	1	13 (68.4)
	≥2	6 (32.6)
Morbidity (*n* = 25)	≤ 30%	12 (48.0)
	30–60%	10 (40.0)
	≥60%	3 (12.0)

*USA, the United States of America; FSS, Functional Status Scale; POPC, Pediatric Overall Performance Category; DDST, Denver Development Screening Test; ABI, acquired brain injury; ARF, acute respiratory failure*.

*^a^Including the Pediatric Evaluation of Disabilities Inventory Computer Adaptive Test (PEDI-CAT), ultrasound, the Movement-Assessment Battery for Children-II (MABC-II), the Patient and Observer Scar Assessment Scale (POSAS), X-ray, the Chalder Fatigue Scale, the Sleep Disturbances Scale, the Medical Research Council (MRC) score*.

*^b^Means during hospitalization and PICU/hospital discharge*.

*^c^One of the following acute brain conditions: traumatic brain injury, cardiac arrest, stroke, brain mass, central nervous system infection or inflammation*.

*^d^Including diseases required continuous kidney replacement therapy (n = 1), hemophagocytic lymphohistiocytosis (n = 1), Bronchiolitis (n = 1), congenital heart disease after open-heart surgery (n = 1)*.

Physical function impairments was reported to have occurred after PICU admission by 10 studies (56.0%) and after PICU/hospital discharge by 11 (44.0%) studies. Six (24%) studies included a follow-up time of >1 year. There were 7 (36.8%) cross-sectional studies and 12 (63.2%) longitudinal studies. The cross-sectional studies reported results over different time periods from admission to long-term post-PICU/hospital discharge. Disabilities in physical function after PICU/hospital discharge (range, 1 month to 10 years) showed that PICU survivors had difficulties in physical function years after PICU discharge. These long-term disabilities widely differed across studies ranging from mild to severe. The course of physical function impairments, from PICU admission to post-PICU/hospital discharge, was reported in 12 studies. The studies investigated the longitudinal trajectory of physical function impairments and demonstrated a considerable and persistent decline in physical function during the PICU stay or post-PICU/hospital discharge.

### Risk Factors for Physical Function Impairments

The risk factors for the impairments noted in the selected studies are defined and categorized in Table 2. A total of 41 risk factors were identified, of which 8 were categorized as personal, 11 as laboratory, and 22 as ICU-related factors. Personal factors included personality, social demographics, and medical history. ICU-related factors included ICU admission, ICU treatment, and patient experience. Three studies reported that older age was related to more physical disabilities (15, 19, 20), and another four studies of PICU survivors showed a negative relationship between younger age and impairments (21–24). Being a woman was a risk factor for impairments in two studies (24, 25), but another study determined that being a man with bronchiolitis was a risk factor (22). Two of the included studies reported that white race had worse outcomes than other races or ethnicities (24, 26).

**Table 2:** Risk factors for physical function impairments.

**Time to assess**	***n* (%, *N* = 25)**	**Categories**	**Risk factors**
Hospitalization/ discharge	11 (44)	Personal	Age, sex, ethnicity, baseline function, the presence of a pre-admission chronic condition
		ICU-related	Unscheduled admission, postoperative admission, surgery in OR, diagnosis, extracorporeal life support, received CPR, neurologic insult, the severity of disease, hospital LOS, days on sedation, MV, weaning achieved
		Laboratory	Brain derived neurotrophic factor, CRP, vascular endothelial growth factor, ALT, AST, prothrombin time, RBS, serum albumin, serum calcium, serum sodium, blood PH
1–3 months after discharge	2 (8)	Personal	Age, sex, ethnicity, the presence of a pre-admission chronic condition, recent trauma
		ICU-related	Source of infection, received CPR, the severity of disease, diagnosis, PICU LOS, hospital LOS, critical care intervention (any), GCS, seizure during admission, discharge to inpatient rehabilitation, electroencephalography
4–6 months after discharge	4 (16)	Personal	Age, ethnicity, baseline function, premature
		ICU-related	Neurologic insult, diagnosis, MV, clonidine, family composition, PICU LOS, hospital LOS, GCS, critical care intervention (any), seizure during admission, discharge to inpatient rehabilitation, electroencephalography
7–12 months after discharge	2 (8)	ICU-related	Parenteral nutrition
Over 1 years after discharge	6 (24)	Personal	Age, maternal education
		ICU-related	DIC score, VAS score, GCS, the severity of disease

*ICU, intensive care unit; OR, operating room; CPR, cardiopulmonary resuscitation; CRP, C-reactive protein; MV, mechanical ventilation; PICU, pediatric intensive care unit; LOS, length of stay; GCS, Glasgow Coma Scale; VAS score, vasopressor score; DIC score, disseminated Intravascular coagulation score; RBS, Random blood sugar; ALT, alanine aminotransferase; AST, aspartate aminotransferase*.

Table 3 presents the most common risk factors reported in >3 studies for physical impairments. The most frequently reported personal risk factors associated with decreased physical function were age, race or ethnicity, a pre-admission chronic condition, and sex; age was observed in 7 (36.8%) studies (15, 19–24). ICU-related determinants were also associated with impairments, including disease severity, duration or the presence of mechanical ventilation (MV), and admission diagnosis. Disease severity was the most reported risk factor in the studies (*n* = 7, 36.8%) (19–21, 23–25, 27), followed by the duration or presence of MV (*n* = 5, 26.3%) (19, 22, 25, 28, 29) and admission diagnosis (*n* = 5, 26.3%) (20, 24, 25, 28, 30).

**Table 3:** The most common risk factors reported in over 3 studies for physical impairments.

**Categories**	***n* (%, *N* = 19)**	**Risk factors**
Personal	7 (36.8)	Age
	3 (15.8)	Sex
	3 (15.8)	Ethnicity
	3 (15.8)	The presence of a pre-admission chronic condition
ICU-related	7 (36.8)	The severity of disease
	5 (26.3)	MV
	5 (26.3)	Admission diagnosis

*ICU, intensive care unit; MV, mechanical ventilation*.

## Discussion

In this review, the risk factors for physical function impairments were systematically collected and summarized. Because PICS has been emphasized since its proposal in 2010 (3), more studies were published in the past 6 years. In addition, more studies have been reported in the United States than in other countries. One possibility is that developed countries have more interest in PICS after critical illness because they have higher ICU survival rates and morbidities. The studies included in this review showed a considerable decline in physical function during the PICU/hospital stay or post-PICU/hospital discharge. The most frequently reported personal risk factors associated with physical function impairments were age, race or ethnicity, sex, the presence of a pre-admission chronic condition, and ICU-related risk factors, including disease severity, duration or the presence of MV, and admission diagnosis.

The physical instruments used in the selected studies varied, and the POPC and FSS were the most frequently used global physical function instruments. The FSS was primarily used during the PICU stay (31–33), and the POPC was used during the PICU stay or post-PICU discharge (19, 20, 22, 24, 28), particularly as a longitudinal measurement. In addition, assessment of muscle wasting and atrophy *via* ultrasound or muscle strength *via* dynamometry were conducted longitudinally during the PICU/hospital stay (25, 29). This review is similar to a recent review by Ong et al. (34), in which diverse measurement tools were used after PICU discharge, such as the Sleep Disturbance Scale for Children (35), the Chalder Fatigue Scale (26), POSAS (21), and MABC-II (36). Due to the complexity and diversity of the current tools, future research should develop a unique and comprehensive instrument to evaluate physical function impairments in PICU survivors. In accordance with the adult PICS field, more research is needed to detect and diagnose physical function impairments (37).

Physical function impairments rates differed across populations, measurement time, and tools used. The impairments rates in a general PICU population ranged from 0.02 to 10.3% at discharge to 38% at 6 months (19, 20, 26). However, the rates of impairments in acquired brain injury patients ranged from 28.9 to 70% (23, 27, 30, 32, 35), 2 to 69% among those with sepsis (19, 21, 24), and 20 to 34.3% among those needing MV (25, 28, 29). Overall, physical function impairments in the included studies ranged from 0.02 to 85.1% during hospitalization/at discharge (15, 19), 20 to 55.7% at 6 months (15, 28), and 2 to 70% at more than 1 year (19, 27). Within the same patient population impairment was lower using POSAS compared with global measures using POPC (19, 21). Furthermore, the longitudinal trajectory of physical function impairments indicated that PICU survivors may experience disabilities years after PICU discharge. Although some long-term improvements have been reported, most physical function levels were lower compared with reference and baseline function. A longitudinal study demonstrated that some impairments resolve with time as acquired impairments decreased from 18% at 3 months to 2% at 1 year following PICU discharge (19). Some studies measured physical function impairments only once, within 6 months after discharge (19, 25, 26, 29, 35). Because impairments may last more than 10 years after discharge (21, 36), more prospective studies are warranted to determine changes in impairments over time by conducting long-term follow-up studies of survivors. Additional important impairments in adult ICU survivors, including sleep disruption and fatigue, seem to be ignored, but data suggest that these problems also occur in children (26, 38, 39).

Physical function impairments in over a third of PICU survivors can present as loss of muscle mass, neuromuscular weakness, impaired lung function, weight loss, and fatigue (17). These impairments restrict daily functioning and can lead to persistent functional disabilities (e.g., limitations in feeding, dressing, and getting out of bed or in mobility) (3, 11, 34). The PICS-p framework is now recognized for children and has standardized follow-up care following a PICU stay, especially for children with a higher risk for significantly worse physical function (11, 17, 40). Early recognition of children at risk for impairments can help prevent these complications by beginning active or passive exercises immediately after admission (41, 42). A total of 41 risk factors were determined in this review. The most common risk factors were reported in more than three studies, but only seven risk factors were defined. Long-term physical function impairments can be persistent, but few studies explored the related risk factors. Future longitudinal research on individual risk factors is needed to develop consensus on physical function impairments.

The most frequently reported personal risk factors associated with decreased physical function were older age, white race, the presence of a pre-admission chronic condition, and being woman. This result is consistent with those of previous studies in adults (43, 44). However, contradictory results were also obtained from some studies. Older age is usually mentioned as a risk factor for physical function impairments in a general PICU population, whereas four of the included studies demonstrated a positive relationship between younger age and impairments among those with specific diagnoses (such as sepsis and acquired brain injury) (21–24). Personal risk factors are not modifiable but can be used to screen for the risk of impairments. The PICS-p framework highlights the baseline status that might possibly impact the health and life of the child for decades (11). A decline in baseline functional status and the presence of pre-admission chronic conditions places the patient at a higher risk for physical function impairments (15, 28, 33, 35). Chronic conditions prior to admission may decrease the patient's baseline function, and it also places the patient at a higher risk of long-term impairments after discharge. In addition, the PICS-p framework emphasizes that family and parents are important for children, but these factors do not seem to have attracted much attention in the currently limited literature (45, 46). In this review, lower maternal education and incomplete family composition were risk factors for physical function impairments (26, 36).

ICU-related determinants, including disease severity, duration or the presence of MV, and admission diagnosis were also associated with decreased levels of physical function. MV during the PICU stay is considered to be one of the main causes leading to ICUAW (47–49). High disease severity, such as an elevated Pediatric Index of Mortality 2 score or Pediatric Risk of Mortality score, and longer duration of MV as risk factors for physical impairments were consistent with the results of the selected studies. Admission diagnosis was also a risk factor for physical function impairments, with trauma and cancer placing the patient at a higher risk compared with other admission diagnoses (20, 24, 28, 30). Although diagnosis at admission and disease severity were unmodifiable factors, they were the key factors in determining the patients' level of physical function and wellbeing (50, 51). Follow-up and rehabilitation programs after PICU discharge focusing on these risk factors might enhance the success of reducing impairments and promote the effective use of health services.

There were several limitations to this review. First, the quality and validity of the included studies were not formally evaluated. Second, some univariate statistical results, but not adjusted statistical results, were accepted. Third, the search term QoL was excluded; thus, some risk factors for impairments may have been missed. Finally, given the limited number of studies and the heterogeneity of the studies (e.g., instruments, population, length of follow-up, design, and outcome measures), it was difficult to calculate and synthesize the data and conclusions. Given such heterogeneity, two of the authors used standardized extraction forms and consensus to determine a more comprehensive evidence summary. This review greatly helps in clearly understanding physical function impairments by integrating studies of risk factors and identifying interventions to prevent or reduce its severity; however, future research is needed.

## Conclusion

In summary, children admitted to the PICU will experience physical function impairments during their PICU stay or post-PICU/hospital discharge, which may last for many years. The most frequently reported risk factors for impairments were age, ethnicity, a pre-admission chronic condition, sex, disease severity, duration or the presence of MV, and admission diagnosis. Of these, the duration of MV is important because it is modifiable. Management of critical care can play a significant role in preventing physical impairments in PICS. It is necessary to develop an objective measurement tool that integrates and assesses multidimensional factors based on an operational definition of physical function impairments. Future studies need to be conducted to reach a consensus on identifying risk factors and mechanisms of physical function impairments in critically ill children.

## Data Availability Statement

The original contributions presented in the study are included in the article/Supplementary Material, further inquiries can be directed to the corresponding author/s.

## Author Contributions

MD: conceptualization, methodology, investigation, and writing—original draft. CY: writing—review and editing, investigation, and software. YL: project administration. All authors contributed to the intellectual content of this manuscript and approved the final manuscript as submitted.

## Funding

This study was funded by the subject named Development and Construction of Polydopamine-doped Calcium Alginate Antimicrobial Hydrogel Dressing and Its Application in Wound Repair (No. CGZHYD202012-006).

## Conflict of Interest

The authors declare that the research was conducted in the absence of any commercial or financial relationships that could be construed as a potential conflict of interest.

## Publisher's Note

All claims expressed in this article are solely those of the authors and do not necessarily represent those of their affiliated organizations, or those of the publisher, the editors and the reviewers. Any product that may be evaluated in this article, or claim that may be made by its manufacturer, is not guaranteed or endorsed by the publisher.
