# Supplementary material for: Risk Factors for Physical Function Impairments in Postintensive Care Syndrome: A Scoping Review
Source: Front Pediatr. 2022 Jun 17;10:905167. doi: 10.3389/fped.2022.905167 (PMC9249083; doi:10.3389/fped.2022.905167)
Supplement: Supplementary file 2 [file Table_2.docx]

**Supplementary Material 2**

**PubMed**

| **Search strategies** | **Results** |
| --- | --- |
| 1. “intensive care units, pediatric” [MeSH] OR (PICU OR ICU OR ((critical* OR intensive*) AND (care* OR ill*))) [Title/Abstract] | 333,431 |
| 1. Child [MeSH] OR infant [MeSH] OR adolescent [MeSH] OR [pediatric OR paediatric OR child* OR adolescen* OR infan* OR preschool* OR teen* OR kindergarten* OR “elementary school*” OR “nursery school*” OR youth* OR baby* OR babies* OR schoolchild* OR toddler*][Title/Abstract] | 4,322,801 |
| 1. "Infant, Newborn"[Mesh] OR (newborn* OR neon* OR "Low Birth Weight" OR Postmature OR Premature OR "Small for Gestational Age" OR VLBW OR LBW) [Title/Abstract] | 929,451 |
| 1. 1 and (2 NOT 3) | 51,041 |
| 1. “postintensive care syndrome” OR PICS OR “ICU syndrome” OR function* OR outcome* OR “follow up” OR disabil* OR physic* OR diaphragm OR myopathy OR neuropathy OR neuromyopathy OR ICUAW OR CIM OR CIP OR CIPNM OR wast* OR weak* OR Frailty OR quadriplegia OR “shortness of breath” OR “quality of life” [Title/Abstract] | 7,878,028 |
| 6. postintensive care syndrome [MeSH] OR Disability Evaluation [MeSH] OR Frailty [MeSH] OR Muscle Weakness [MeSH] OR Paresis [MeSH] | 76,526 |
| 7. 5 OR 6 | 7,897,680 |
| 8. 4 AND 7 | 24765 |
| 9. ["Case Reports" OR "Review"][Publication Type] | 4,951,906 |
| 10. 8 NOT 9 | 20,271 |
| 11. Animals [MeSH Terms] AND Animals[Title/Abstract] | 24,690,000 |
| 12. 10 NOT 11 | 2,784 |

**Cochrane Library**

| **Search strategies** | **Results** |
| --- | --- |
| 1. “intensive care units, pediatric” [MeSH] OR (PICU OR ICU OR ((critical* OR intensive*) AND (care* OR ill*))) [ti,ab,kw] | 48540 |
| 1. Child [MeSH] OR infant [MeSH] OR adolescent [MeSH] OR [pediatric OR paediatric OR child* OR adolescen* OR infan* OR preschool* OR teen* OR kindergarten* OR “elementary school*” OR “nursery school*” OR youth* OR baby* OR babies* OR schoolchild* OR toddler*][ti,ab,kw] | 300418 |
| 1. "Infant, Newborn"[Mesh] OR (newborn* OR neon* OR "Low Birth Weight" OR Postmature OR Premature OR "Small for Gestational Age" OR VLBW OR LBW) [ti,ab,kw] | 50709 |
| 1. 1 and ( 2 NOT 3 ) | 6776 |
| 1. “postintensive care syndrome” OR PICS OR “ICU syndrome” OR function* OR outcome* OR “follow up” OR disabil* OR physic* OR diaphragm OR myopathy OR neuropathy OR neuromyopathy OR ICUAW OR CIM OR CIP OR CIPNM OR wast* OR weak* OR Frailty OR quadriplegia OR “shortness of breath” OR “quality of life” [ti,ab,kw] | 957594 |
| 1. Disability Evaluation [MeSH] OR Frailty [MeSH] OR Muscle Weakness [MeSH] OR Paresis [MeSH] | 5280 |
| 7. 5 OR 6 | 967684 |
| 8. 4 AND 7 | 5048 |
| 1. Animal*[ti,ab,kw] | 31714 |
| 10. 8 NOT 9 | 4978 |
| 11. Trails | 3553 |

**Embase**

| **Search strategies** | **Results** |
| --- | --- |
| 1. pediatric intensive care unit [MeSH] OR (picu OR icu OR ((critical* OR intensive*) AND (care* OR ill*))) [ti,ab,kw] | 549813 |
| 2. (Child [MeSH] OR infant [MeSH] OR adolescent [MeSH] OR [pediatric OR paediatric OR child* OR adolescen* OR infan* OR preschool* OR teen* OR kindergarten* OR “elementary school*” OR “nursery school*” OR youth* OR baby* OR babies* OR schoolchild* OR toddler*][ti,ab,kw]) | 4850004 |
| 3. 1 AND 2 | 130782 |
| 4. (“postintensive care syndrome” OR PICS OR “ICU syndrome” OR function* OR outcome* OR disabil* OR physic* OR diaphragm OR myopathy OR neuropathy OR neuromyopathy OR ICUAW OR CIM OR CIP OR CIPNM OR “muscle wast*” OR “muscle weak*” OR quadriplegia OR “quality of life”) [ti,ab,kw] | 12698912 |
| 5.Disability [MeSH] OR physical disease [MeSH] OR Paresis [MeSH] | 24176533 |
| 6. 1. 4 OR 5 | 28375249 |
| 7. 1. 3 AND 6 | 116672 |
| 8. Risk* [ti,ab,kw] OR risk [MeSH] | 4227804 |
| 9. 7 AND 8 | 36456 |

**Web of Science**

| **Search strategies** | **Results** |
| --- | --- |
| 1. Pediatric intensive care unit OR picu OR icu OR ((critical* OR intensive*) AND (care* OR ill*)) | 454247 |
| 1. pediatric OR paediatric OR child* OR adolescen* OR infan* OR preschool* OR teen* OR kindergarten* OR “elementary school*” OR “nursery school*” OR youth* OR baby* OR babies* OR schoolchild* OR toddler* | 2737374 |
| 1. 1 and 2 | 77224 |
| 1. “postintensive care syndrome” OR PICS OR “ICU syndrome” OR function* OR outcome* OR disabil* OR physic* OR myopathy OR neuropathy OR neuromyopathy OR ICUAW OR CIM OR CIP OR CIPNM OR wast* OR weak* OR Frailty | 12997155 |
| 1. 3 AND 4 | 36882 |
| 1. “risk factor*” | 899990 |
| 1. 5 AND 6 | 4716 |
